# Supplementary material for: Resolving the full spectrum of human genome variation using Linked-Reads
Source: Genome Res. 2019 Apr;29(4):635–45. doi: 10.1101/gr.234443.118 (PMC6442396; doi:10.1101/gr.234443.118)
Supplement: Supplemental Material [file supp_gr.234443.118_Supplemental_longranger-2.2.zip › longranger-2.2.tar.bz2!/lr2.2-public/lib/stan/cmdstan/stan/doc/api/html/ordered__logistic_8hpp_source.html]

Stan: src/stan/prob/distributions/univariate/discrete/ordered\_logistic.hpp Source File


|  |  |
| --- | --- |
|  | Stan  2.5.0  probability, sampling & optimization |


- Main Page
- Related Pages
- Namespaces
- Classes
- Files

- File List
- File Members

All Classes Namespaces Files Functions Variables Typedefs Enumerations Enumerator Friends Macros Pages

- src
- stan
- prob
- distributions
- univariate
- discrete

ordered\_logistic.hpp

Go to the documentation of this file.

1 #ifndef STAN\_\_PROB\_\_DISTRIBUTIONS\_\_UNIVARIATE\_\_DISCRETE\_\_ORDERED\_LOGISTIC\_HPP

2 #define STAN\_\_PROB\_\_DISTRIBUTIONS\_\_UNIVARIATE\_\_DISCRETE\_\_ORDERED\_LOGISTIC\_HPP

3

4 #include <boost/random/uniform\_01.hpp>

5 #include <boost/random/variate\_generator.hpp>

6 #include <stan/prob/distributions/multivariate/discrete/categorical.hpp>

7

8 #include <stan/prob/traits.hpp>

9 #include <stan/math/error\_handling.hpp>

10 #include <stan/math/functions/inv\_logit.hpp>

11 #include <stan/math/functions/log1m.hpp>

12 #include <stan/math/functions/log1m\_exp.hpp>

13 #include <stan/math/functions/log1p\_exp.hpp>

14 #include <stan/math/matrix\_error\_handling.hpp>

15 #include <stan/math/error\_handling.hpp>

16 #include <stan/prob/constants.hpp>

17

18

19 namespace stan {

20

21  namespace prob {

22

23  template <typename T>

24  inline T log\_inv\_logit\_diff(const T& alpha, const T& beta) {

25  using std::exp;

26  using stan::math::log1m\_exp;

27  using stan::math::log1p\_exp;

28  return beta + log1m\_exp(alpha - beta) - log1p\_exp(alpha) - log1p\_exp(beta);

29  }

30

31  // y in 0,...,K-1; c.size()==K-2, c increasing, lambda finite

58  template <bool propto, typename T\_lambda, typename T\_cut>

59  typename boost::math::tools::promote\_args<T\_lambda,T\_cut>::type

60  ordered\_logistic\_log(int y, const T\_lambda& lambda,

61  const Eigen::Matrix<T\_cut,Eigen::Dynamic,1>& c) {

62  using std::exp;

63  using std::log;

64  using stan::math::inv\_logit;

65  using stan::math::log1m;

66  using stan::math::log1p\_exp;

67

68  static const char\* function = "stan::prob::ordered\_logistic(%1%)";

69

70  using stan::math::check\_finite;

71  using stan::math::check\_positive;

72  using stan::math::check\_nonnegative;

73  using stan::math::check\_less;

74  using stan::math::check\_less\_or\_equal;

75  using stan::math::check\_greater;

76  using stan::math::check\_bounded;

77

78  int K = c.size() + 1;

79

80  typename boost::math::tools::promote\_args<T\_lambda,T\_cut>::type lp(0.0);

81  check\_bounded(function, y, 1, K, "Random variable", &lp);

82  check\_finite(function, lambda, "Location parameter", &lp);

83  check\_greater(function, c.size(), 0, "Size of cut points parameter", &lp);

84  for (int i = 1; i < c.size(); ++i)

85  check\_greater(function, c(i), c(i - 1), "Cut points parameter", &lp);

86

87  check\_finite(function, c(c.size()-1), "Cut points parameter", &lp);

88  check\_finite(function, c(0), "Cut points parameter", &lp);

89

90  // log(1 - inv\_logit(lambda))

91  if (y == 1)

92  return -log1p\_exp(lambda - c(0));

93

94  // log(inv\_logit(lambda - c(K-3)));

95  if (y == K) {

96  return -log1p\_exp(c(K-2) - lambda);

97  }

98

99  // if (2 < y < K) { ... }

100  // log(inv\_logit(lambda - c(y-2)) - inv\_logit(lambda - c(y-1)))

101  return log\_inv\_logit\_diff(c(y-2) - lambda,

102  c(y-1) - lambda);

103

104  }

105

106  template <typename T\_lambda, typename T\_cut>

107  typename boost::math::tools::promote\_args<T\_lambda,T\_cut>::type

108  ordered\_logistic\_log(int y, const T\_lambda& lambda,

109  const Eigen::Matrix<T\_cut,Eigen::Dynamic,1>& c) {

110  return ordered\_logistic\_log<false>(y,lambda,c);

111  }

112

113  template <class RNG>

114  inline int

115  ordered\_logistic\_rng(const double eta,

116  const Eigen::Matrix<double,Eigen::Dynamic,1>& c,

117  RNG& rng) {

118  using boost::variate\_generator;

119  using stan::math::inv\_logit;

120

121  static const char\* function = "stan::prob::ordered\_logistic(%1%)";

122

123  using stan::math::check\_finite;

124  using stan::math::check\_positive;

125  using stan::math::check\_nonnegative;

126  using stan::math::check\_less;

127  using stan::math::check\_less\_or\_equal;

128  using stan::math::check\_greater;

129  using stan::math::check\_bounded;

130

131  check\_finite(function, eta, "Location parameter", (double\*)0);

132  check\_greater(function, c.size(), 0, "Size of cut points parameter",

133  (double\*)0);

134  for (int i = 1; i < c.size(); ++i) {

135  check\_greater(function, c(i), c(i - 1),

136  "Cut points parameter", (double\*)0);

137  }

138  check\_finite(function, c(c.size()-1),

139  "Cut points parameter", (double\*)0);

140  check\_finite(function, c(0),

141  "Cut points parameter", (double\*)0);

142

143  Eigen::VectorXd cut(c.rows()+1);

144  cut(0) = 1 - inv\_logit(eta - c(0));

145  for(int j = 1; j < c.rows(); j++)

146  cut(j) = inv\_logit(eta - c(j - 1)) - inv\_logit(eta - c(j));

147  cut(c.rows()) = inv\_logit(eta - c(c.rows() - 1));

148

149  return stan::prob::categorical\_rng(cut, rng);

150  }

151  }

152 }

153

154 #endif

log1m\_exp.hpp

stan::math::check\_bounded

bool check\_bounded(const char \*function, const T\_y &y, const T\_low &low, const T\_high &high, const char \*name, T\_result \*result)

**Definition:** check\_bounded.hpp:79

stan::agrad::log1p\_exp

fvar< T > log1p\_exp(const fvar< T > &x)

**Definition:** log1p\_exp.hpp:15

stan::math::log1p\_exp

boost::math::tools::promote\_args< T >::type log1p\_exp(const T a)

Calculates the log of 1 plus the exponential of the specified value without overflow.

**Definition:** log1p\_exp.hpp:44

stan::math::check\_greater

bool check\_greater(const char \*function, const T\_y &y, const T\_low &low, const char \*name, T\_result \*result)

**Definition:** check\_greater.hpp:58

traits.hpp

log1p\_exp.hpp

error\_handling.hpp

stan::math::check\_finite

bool check\_finite(const char \*function, const T\_y &y, const char \*name, T\_result \*result)

Checks if the variable y is finite.

**Definition:** check\_finite.hpp:52

stan::math::inv\_logit

boost::math::tools::promote\_args< T >::type inv\_logit(const T a)

Returns the inverse logit function applied to the argument.

**Definition:** inv\_logit.hpp:52

matrix\_error\_handling.hpp

constants.hpp

stan::prob::categorical\_rng

int categorical\_rng(const Eigen::Matrix< double, Eigen::Dynamic, 1 > &theta, RNG &rng)

**Definition:** categorical.hpp:131

stan::prob::ordered\_logistic\_log

boost::math::tools::promote\_args< T\_lambda, T\_cut >::type ordered\_logistic\_log(int y, const T\_lambda &lambda, const Eigen::Matrix< T\_cut, Eigen::Dynamic, 1 > &c)

Returns the (natural) log probability of the specified integer outcome given the continuous location ...

**Definition:** ordered\_logistic.hpp:60

stan::agrad::inv\_logit

fvar< T > inv\_logit(const fvar< T > &x)

**Definition:** inv\_logit.hpp:15

log1m.hpp

stan::prob::log\_inv\_logit\_diff

T log\_inv\_logit\_diff(const T &alpha, const T &beta)

**Definition:** ordered\_logistic.hpp:24

stan::math::check\_nonnegative

bool check\_nonnegative(const char \*function, const T\_y &y, const char \*name, T\_result \*result)

**Definition:** check\_nonnegative.hpp:52

stan::agrad::log1m\_exp

fvar< T > log1m\_exp(const fvar< T > &x)

**Definition:** log1m\_exp.hpp:17

stan::math::check\_positive

bool check\_positive(const char \*function, const T\_y &y, const char \*name, T\_result \*result)

**Definition:** check\_positive.hpp:54

stan::math::check\_less\_or\_equal

bool check\_less\_or\_equal(const char \*function, const T\_y &y, const T\_high &high, const char \*name, T\_result \*result)

**Definition:** check\_less\_or\_equal.hpp:52

stan::math::log1m\_exp

boost::math::tools::promote\_args< T >::type log1m\_exp(const T a)

Calculates the log of 1 minus the exponential of the specified value without overflow log1m\_exp(x) = ...

**Definition:** log1m\_exp.hpp:40

inv\_logit.hpp

categorical.hpp

stan::math::check\_less

bool check\_less(const char \*function, const T\_y &y, const T\_high &high, const char \*name, T\_result \*result)

**Definition:** check\_less.hpp:52

stan::prob::ordered\_logistic\_rng

int ordered\_logistic\_rng(const double eta, const Eigen::Matrix< double, Eigen::Dynamic, 1 > &c, RNG &rng)

**Definition:** ordered\_logistic.hpp:115

stan::agrad::log

fvar< T > log(const fvar< T > &x)

**Definition:** log.hpp:15

stan::agrad::exp

fvar< T > exp(const fvar< T > &x)

**Definition:** exp.hpp:16

stan::math::log1m

boost::math::tools::promote\_args< T >::type log1m(T x)

Return the natural logarithm of one minus the specified value.

**Definition:** log1m.hpp:40


---

[ Stan Home Page ]

*© 2011–2014,
Stan Development Team.*
